# Deformation behaviors of peat with influence of organic matter

**DOI:** 10.1186/s40064-016-2232-3

**Published:** 2016-05-10

**Authors:** Min Yang, Kan Liu

**Affiliations:** Department of Geotechnical Engineering, Tongji University, Siping Road No. 1239, Shanghai, 200092 China; Key Laboratory of Geotechnical and Underground Engineering of Ministry of Education, Tongji University, Shanghai, 200092 China

**Keywords:** Peat, Compressibility of organic matter, Entrapped gas bubbles, Consolidation model

## Abstract

Peat is a kind of special material rich in organic matter. Because of the high content of organic matter, it shows different deformation behaviors from conventional geotechnical materials. Peat grain has a non-negligible compressibility due to the presence of organic matter. Biogas can generate from peat and can be trapped in form of gas bubbles. Considering the natural properties of peat, a special three-phase composition of peat is described which indicates the existence of organic matter and gas bubbles in peat. A stress–strain–time model is proposed for the compression of organic matter, and the surface tension effect is considered in the compression model of gas bubbles. Finally, a mathematical model has been developed to simulate the deformation behavior of peat considering the compressibility of organic matter and entrapped gas bubbles. The deformation process is the coupling of volume variation of organic matter, gas bubbles and water drainage. The proposed model is used to simulate a series of peat laboratory oedometer tests, and the model can well capture the test results with reasonable model parameters. Effects of model parameters on deformation of peat are also analyzed.

## Background

Peat is a kind of engineering material rich in organic matter. It has a widely distribution around the world and shows unique compression properties. Because of the complicated physical composition of peat, it has been recognized that the deformation of this material is extremely complex. Peat may undergo an axial strain as large as 50 % due to the highly compressible property of natural deposits (Berry and Poskitt 1972). The textures of peat natural deposits and the high content of organic matter have significant effects on the deformation behavior of peat.

Under appropriate climatic and topographic conditions, organic matter in peat is derived from vegetation that has been chemically changed and fossilized (Dhowian and Edil 1980). Minerals or solid phase are usually considered incompressible in soil, but it may not be appropriate for peat with high organic matter content. The organic matter phase or peat grains could be compressible, which could be an important factor effecting deformation properties of peat. Although some researchers have noticed this (Bery and Vickers 1975; Robinson 2003), no similar studies have been done to consider this point of view in peat. It’s necessary to understand how organic matter affects the deformation process of peat.

With the high content of organic matter, another feature of peat is that biogas (e.g., methane) can generate from its natural deposits. During generation and migration, biogas can be trapped in the micro voids of peat as small gas bubbles. For materials with gas bubble entrapment, the deformation behaviors and other mechanical properties are different from traditional unsaturated conditions where gas phase is assumed connected (Wheeler 1988; Sills et al. 1991). Materials containing gas bubbles are considered as a special type of engineering materials and are usually considered as “quasi-saturated” (Faybishenko 1995). In these materials, gas phase could be present as isolated bubbles once the water saturation degree is larger than 85 % (Sparks 1963). Studies have shown that gas bubbles present in offshore soils due to the decomposition of sedimentary organic matter (Whelan et al. 1975). With the present of gas bubbles, immediate undrained compressions have been found in gassy soils (Nageswaran 1983). Thus, the existence of entrapped gas bubbles within peat can exert a significant influence on the properties and deformation behaviors of peat.

Terzaghi’s one-dimensional consolidation theory has been widely used in the deformation problem for porous materials. Some researchers have extended the consolidation theory by considering the compressibility of solid phase and the existence of gas phase (Skempton 1961; Fredlund and Hasan 1979; Lade and De Boer 1997). In most cases, the consolidation theory is used for mineral materials like soil. The deformation behavior of peat may not be well-characterized by the traditional one dimensional consolidation theory due to the high organic matter content and gas bubble entrapment.

Some unique consolidation behaviors (e.g., large deformation, immediate settlement after loading and low permeability) have been observed in peat (Berry and Poskitt 1972; Long and Boylan 2013; Lee et al. 2015). It is thus important to propose an appropriate consolidation model to describe the mechanical characteristics and the deformation behavior of peat. In this paper, a consolidation model is proposed for peat under the three phase composition of this material. The model considers the compressibility of organic matter and entrapped gas bubbles in peat. The deformation of organic matter is described by a stress and time dependent empirical model. The mechanical properties of entrapped gas bubbles are studied as ideal gas. The proposed consolidation model is applied on a set of peat oedometer tests and the model can well describe the one dimensional consolidation behavior of peat.

## Basic descriptions of peat

### High in organic matter content

Peat is formed by the gradual accumulation of plant remnants, and the natural organic matter content in peat is high and variable. It has been recognized that the presence of organic matter has significant effects on engineering properties of peat. With high content of organic matter, peat exhibits high water content, large void ratio and low bulk density. Except for the difference of these conventional index properties, the organic matter itself may show some unique properties, for example compressible. Bery and Vickers (1975) have mentioned that the peat particles themselves may be compressible in their study on fibrous peat consolidation. Robinson (2003) indicated the organic matrix is compressible, which gives wrong interpretation of the primary consolidation by Terzaghi’s theory. It might be inappropriate that study on peat is still based on ideas or methods for mineral soils. An obvious initial deformation appears when loading on peat samples, which could be partly caused by organic matter compression. But no detailed studies on compressibility of organic matter in peat have been found. This may be because natural organic matter is in different forms and the structure is very complex. Usually it’s difficult even impossible to quantify the effects of organic matter on peat through a controlled experiment (Choo et al. 2015). We attempt to simulate the compression of organic matter in peat by presenting a unified empirical model. In fact, similar properties have been found in Victorian brown coal from the author’s preliminary studies (Liu et al. 2014a). Victorian brown coal is a kind of intermediate geotechnical materials (IGMs) fossilized from peat after a long time of coalification process (Hayashi and Li 2004). The form of the organic matter compression model is proposed by taking reference from some empirical creep models of soil. The following empirical equation is normally used to describe the stress and time dependent deformation behavior of soils:

1 $$\varepsilon = f(\sigma ,t) = f_{1} (\sigma )f_{2} (t)$$

in which *f*_1_(*σ*) is to describe stress related deformation and *f*_2_(*t*) is the time related deformation. Some researchers studied the creep behavior of soil based on the idea of Eq. (1) and empirical models had been proposed (Singh and Mitchell 1968; Mesri et al. 1981; Lin and Wang 1998). In these models, the stress–strain function (*f*_1_(*σ*)) varies but the strain–time function (*f*_2_(*t*)) usually takes the form of exponential equations. The authors propose an initial constrained modulus of organic matter *E*_*m*_ to describe the stress–strain function (*f*_1_(*σ*)), and still use the exponential form for the strain–time function (*f*_2_(*t*)). Then a simple unified stress–strain–time model is proposed here to describe the compressibility of organic matter in peat under one-dimensional compression:

2 $$\varepsilon_{m} = \left( {\frac{1}{{E_{m} }}\sigma } \right)\left( {\frac{t}{{t_{1} }}} \right)^{\lambda }$$

where, *ε*_*m*_ is strain of organic matter; *σ* is applied vertical total stress; *t*_1_ is unit time; *E*_*m*_ is initial constrained modulus of organic matter; *λ* is time factor.

### Gas bubble entrapment

There are many conditions that engineering materials are not fully saturated and the voids are filled partly with water and partly with gas. Under the condition of a high degree of saturation, the gas phase is discontinuous and is in the form of discrete bubbles (Wheeler 1988). Due to the decomposition process, organic matter in peat can be converted into gases including carbon dioxide and methane. Under near saturated conditions, these gases accumulate into bubbles that remain trapped within peat deposit (Pichan and O’Kelly 2012). The mechanism of entrapped gas bubbles is extremely complicated. Basically the deformation of gas bubbles is controlled by pore air pressure *u*_*g*_ from equation of Boyle’s law. Considering the surface tension effect between gas bubbles and water, the gas bubble pressure *u*_*g*_ is not equal to water pressure *u*_*w*_ in the material. Usually, the difference between the air pressure *u*_*g*_ and the water pressure *u*_*w*_ can be computed by Eq. (3) considering the equilibrium of gas bubbles with radius *r* (Schuurman 1966; Wheeler 1988):

3 $$u_{g} - u_{w} = 2q/r$$

where *q* is the surface tension and *r* is the radius of gas bubbles.

The temperature is considered constant during tests, the surface tension *q* is dependent on the temperature, therefore *q* is constant as well. And the diminution of surface tension *q* with increasing air pressure can be neglected as discussed by Schuurman (1966), so a constant value (7.4 × 10^−3^ N/m) of *q* is used in the paper.

Entrapped gas could be exist as the form of small bubbles compared with average particle size or large gas voids. Wheeler (1988) and Pietruszczak and Pande (1996) discussed the difference between the two kinds of gassy soils. When the gas bubbles are small compared with peat particle size, the bubbles fit within the normal void spaces and the radius of curvature of gas–water interface is equal to the radius *r* of the bubble. At the opposite extreme, gas bubbles are much larger than peat particle size, which generates a large gas-filled void. Then the gas–water interfaces are formed by lots of small menisci which bridge the gaps between the particles. The radius of curvature of these menisci is not necessarily equal to the radius *r* of the bubble. As a simplification, the size of small gas bubbles is assumed to be trapped within the voids of peat grains.

Electron microscope scanning tests of peat from different places have been carried out by some researchers. Lv et al. (2011) obtained the results that the average void diameter is about 10 μm and the large void is up to 25 μm diameter for peat samples from northern east China. Xiong (2005) and Liu et al. (2014b) got the average void diameter is about 13.65 μm for peat samples from Kunming. A void diameter range of 3–20 μm is obtained by Wang (2013)and their tested peat samples are from Hangzhou, east China. Considering the void size of peat, the radius *r* of gas bubbles can be determined and it should be smaller than void sizes. An average initial radius *r*_0_ of gas bubbles are used in the following case studies.

Without considering gas dissolution and exsolution, the gas phase in peat is considered as ideal gas and the deformation obeys Boyle’s law (Schuurman 1966), which is:

4 $$(P_{a} + 2q/r_{0} )V_{g0} = (P_{a} + 2q/r + u_{w} )V_{g}$$

where *V*_*g*_ is gas volume in peat and *r* is the radius of gas bubbles, the subscript *0* represents the initial value of each parameter; *P*_*a*_ is the atmospheric pressure.

### Three phase composition of peat

**Fig. 1:**
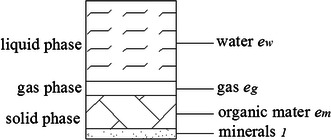
Three phases of peat

Peat has complex textures and physical composition, and peat solid phase can be considered as a mixture of organic matter and minerals. Even under conventional saturated condition, small gas bubbles can also be trapped within the voids of peat grains. Landva and Pheeney (1980) described the characteristics of three phase peat based on a series of electron microscope scanning tests. The author also proposed a similar conceptual model on Victorian brown coal which is a kind of organic material fossilized from peat (Liu et al. 2014a). Based on discussions of above sections, we know that peat can be considered as a three phase mixture and in the state of quasi-saturated. Especially, the solid phase of peat should be divided into mineral part and organic matter part. A schematic diagram of the special three phase composition of peat is shown in Fig. 1. Some parameter definitions and assumptions are made as follows.

As usual analysis method of one dimensional consolidation theory, a representative element with unit volume *dxdydz* is taken here to do the analysis. The unit element is assumed to satisfy both the basic assumptions of Terzaghi’s theory and assumptions in above discussions. The special three phase composition as shown in Fig. 1 is adopted for the unit element. Similar to the definition of conventional void ratio *e*, some parameter definitions are made as follow.

5 $$e_{m} = \frac{{V_{m} }}{{V_{s} }}\quad e_{g} = \frac{{V_{g} }}{{V_{s} }}\quad e_{w} = \frac{{V_{w} }}{{V_{s} }}$$

6 $$a = e_{m} + e_{g} + e_{w}$$

where, *e*_*w*_, *e*_*g*_ and *e*_*m*_ are defined as volume ratios of water, gas and organic matter, respectively; *V*_*w*_, *V*_*g*_, *V*_*m*_ and *V*_*s*_ are water volume, gas volume, organic matter volume and mineral volume, respectively; and *a* is the ratio of the changeable volume to the volume of the incompressible solid minerals, which is the sum of *e*_*w*_, *e*_*g*_ and *e*_*m*_.

Usually the basic geotechnical indexes of peat are known quantities including density of peat (*ρ*), water content (*ω*), organic matter content (*ω*_*m*_) and unit weight of peat solid phase (*γ*_*p*_), then above defined parameters can be calculated with mass and volume conservation for a certain peat sample.

Under above definitions, conventional void ratio *e* can be calculated as:

7 $$e = \frac{{e_{g} + e_{w} }}{{1 + e_{m} }}$$

A parameter *β* is defined as the ratio of initial organic matter volume (*V*_*m0*_) to initial total volume (*V*_*0*_), that is:

8 $$\beta = \frac{{V_{m0} }}{{V_{0} }} = \frac{{e_{m0} }}{{1 + a_{0} }}$$

The gas volume content is defined as:

9 $$S_{g} = \frac{{V_{g} }}{V} = \frac{{e_{g} }}{1 + a}$$

where, *V* is the total volume.

## Mathematic model of peat

Based on above assumptions and parameter definitions, a mathematical model for peat deformation is derived with considering the compression of organic matter and entrapped gas bubbles.

Also for the unit element, volumetric continuity in the element is:

10 $$\Delta V_{m} +\Delta V_{g} +\Delta V_{w} =\Delta V_{c}$$

where, Δ*V*_*c*_ is the change of volume during compression, due to the volume change of organic matter Δ*V*_*m*_, gas Δ*V*_*g*_ and water Δ*V*_*w*_.

During a short time *dt*:

11 $$\frac{{dV_{w} }}{dt} = \frac{{dV_{c} }}{dt} - \frac{{dV_{m} }}{dt} - \frac{{dV_{g} }}{dt}$$

As the basic Terzaghi’s theory, assuming water flow in the materials obeys Darcy’s law and hydraulic conductivity *k* keeps constant during the short time period, in the unit element with the dimension of *dx, dy* and *dz*, the water volume increment in the element during time *dt* is same with Terzaghi’s theory:

12 $$\frac{dQ}{dt} = \frac{k}{{\gamma_{w} }}\frac{{\partial^{2} u}}{{\partial z^{2} }}dxdydz$$

where *u* is the excess pore water pressure; and *γ*_*w*_ is the unit weight of water.

Water flow follows the mass of conservation:

13 $$\frac{{dV_{w} }}{dt} = \frac{dQ}{dt}$$

Based on above constitutive relations, the finial mathematical equation can be obtained. But the three parts *dV*_c_/*dt*, *dV*_m_/*dt* and *dV*_g_/*dt* in the right side of Eq. (11) need to be determined respectively.

Considering the parameter definitions, the defined parameter *a* has the similar situation of conventional void ratio *e*. In basic derivation of Terzaghi’s theory:

14 $$\frac{{dV_{c} }}{dt} = \frac{1}{1 + e}\frac{\partial e}{\partial t}dxdydz$$

Similarly, we have:

15 $$\frac{{dV_{c} }}{dt} = \frac{1}{1 + a}\frac{\partial a}{\partial t}dxdydz$$

Considering the principle of effective stress,

16 $$\sigma^{\prime} = \sigma - u$$

17 $$\frac{\partial a}{\partial t} = \frac{\partial a}{{\partial \sigma^{{\prime }} }}\frac{{\partial \sigma^{{\prime }} }}{\partial t} = \frac{\partial a}{{\partial \sigma^{{\prime }} }}\left( {\frac{\partial \sigma }{\partial t} - \frac{\partial u}{\partial t}} \right)$$

In Eq. (17), the calculation of *∂a/∂σ*^′^ is similar to *∂e/∂σ*^′^ (compression coefficient *c*) of Terzaghi’s theory. For a certain peat sample, the value is calculated by the total increment Δ*a/*Δ*σ*^′^ in the whole consolidation process.

The other two parts *dV*_m_/*dt* and *dV*_g_/*dt* in Eq. (11) represent the compression behaviors of organic matter and entrapped gas bubbles respectively, which can be determined based on the discussions in above sections.

For the compression of organic matter in peat, at any time *t*, the volume of organic matter can be expressed with the initial organic matter volume *V*_*m*0_ and strain *ε*_*m*_,

18 $$V_{m} = V_{m0} (1 - \varepsilon_{m} )$$

Then

19 $$\frac{{dV_{m} }}{dt} = - V_{m0} \frac{{d\varepsilon_{m} }}{dt}$$

Based on stress–strain–time Eq. (2) and definition of parameter *β*, we have:

20 $$\frac{{dV_{m} }}{dt} = - \beta \frac{1}{{E_{m} }}\left( {\frac{\partial \sigma }{\partial t}t^{\lambda } + \lambda \sigma t^{\lambda - 1} } \right)dxdydz$$

From Eq. (4) and parameter definitions in Eq. (5), the increment of gas volume during a short time *dt* in the unit element can be calculated as:

21 $$\frac{{dV_{g} }}{dt} = - \frac{{(P_{a} + 2q/r_{0} )V_{g0} }}{{(P_{a} + 2q/r + u)^{2} }}\frac{d(2q/r + u)}{dt} = - \frac{{V_{g} }}{{P_{a} + 2q/r + u}}\frac{d(2q/r + u)}{dt}$$

22 $$V_{g} = \frac{{e_{g} }}{1 + a}dxdydz$$

23 $$\frac{{dV_{g} }}{dt} = - \frac{{e_{g} }}{{(1 + a)(P_{a} + 2q/r + u)}}\frac{\partial (2q/r + u)}{\partial t}dxdydz$$

Substituting Eqs. (15), (20), (23) into (13), the consolidation model of peat with compressible organic matter and gas bubbles can be described as:

24 $$\begin{aligned} \frac{k}{{\gamma_{w} }}\frac{{\partial^{2} u}}{{\partial z^{2} }} & = \frac{1}{{(1 + e)(1 + e_{m} )}}\frac{\partial a}{{\partial \sigma^{\prime} }}\left(\frac{\partial \sigma }{\partial t} - \frac{\partial u}{\partial t}\right) + \beta \frac{1}{{E_{m} }}\left(\frac{\partial \sigma }{\partial t}t^{\lambda } + \lambda \sigma t^{\lambda - 1} \right) \\ & \quad + \frac{{e_{g} }}{{(1 + e)(1 + e_{m} )(P_{a} + 2q/r + u)}}\left( - \frac{2q}{{r^{2} }}\frac{\partial r}{\partial t} + \frac{\partial u}{\partial t}\right) \\ \end{aligned}$$

One-dimensional finite difference form of Eq. (24) is employed to solve this equation in Excel. By fitting the numerical and experimental consolidation curves, the compressibility of organic matter and gas content can be determined. The model is applied on a set of historical consolidation data of peat in the following sections. The calculation results agree well with test results in each case study.

## Model application on peat oedometer Tests

### Case 1: Peat from northern east of China

Five typical peat samples are used from a set of one dimensional consolidation tests reported by Lv et al. (2011). The 5 peat samples are from northern east of China and at different organic matter content. The peat sample size is conventional, which is 79.8 mm in diameter and 20 mm in height. A vertical load of 50 kPa is applied on each sample. Table 1 shows the basic properties of the 5 samples. In the table, the organic content *ω*_*m*_ is the ratio of the mass of organic matter to the mass of total solid matter, *ρ* is the density of peat, *γ*_*p*_ is unit weight of solid matter, *ω* is water content, *e* is void ratio. The basic indexes are used to calculate the model parameters *e*_*g*_, *e*_*m*_ and *e*_*w*_ as defined in above sections. Lv et al. (2011) found that the samples present different compression behaviours with the increase of organic matter content. The authors notice that there is almost a liner relationship between time and settlement in the first few minutes, and that part of the settlement attributes to a large percentage of the total settlement.

**Table 1:** Properties of peat samples in case 1

Sample no.	*ρ* (g/cm^3^)	*γ*_*p*_ (kN/m^3^)	*ω*_*m*_ (%)	*ω* (%)	*e*
1	1.122	19.24	36.34	117.01	2.721
2	1.145	19.20	43.65	142.65	3.069
3	1.045	18.81	55.99	272.24	5.722
4	1.010	16.90	69.29	385.00	7.115
5	0.944	16.58	85.36	467.55	8.968

**Fig. 2:**
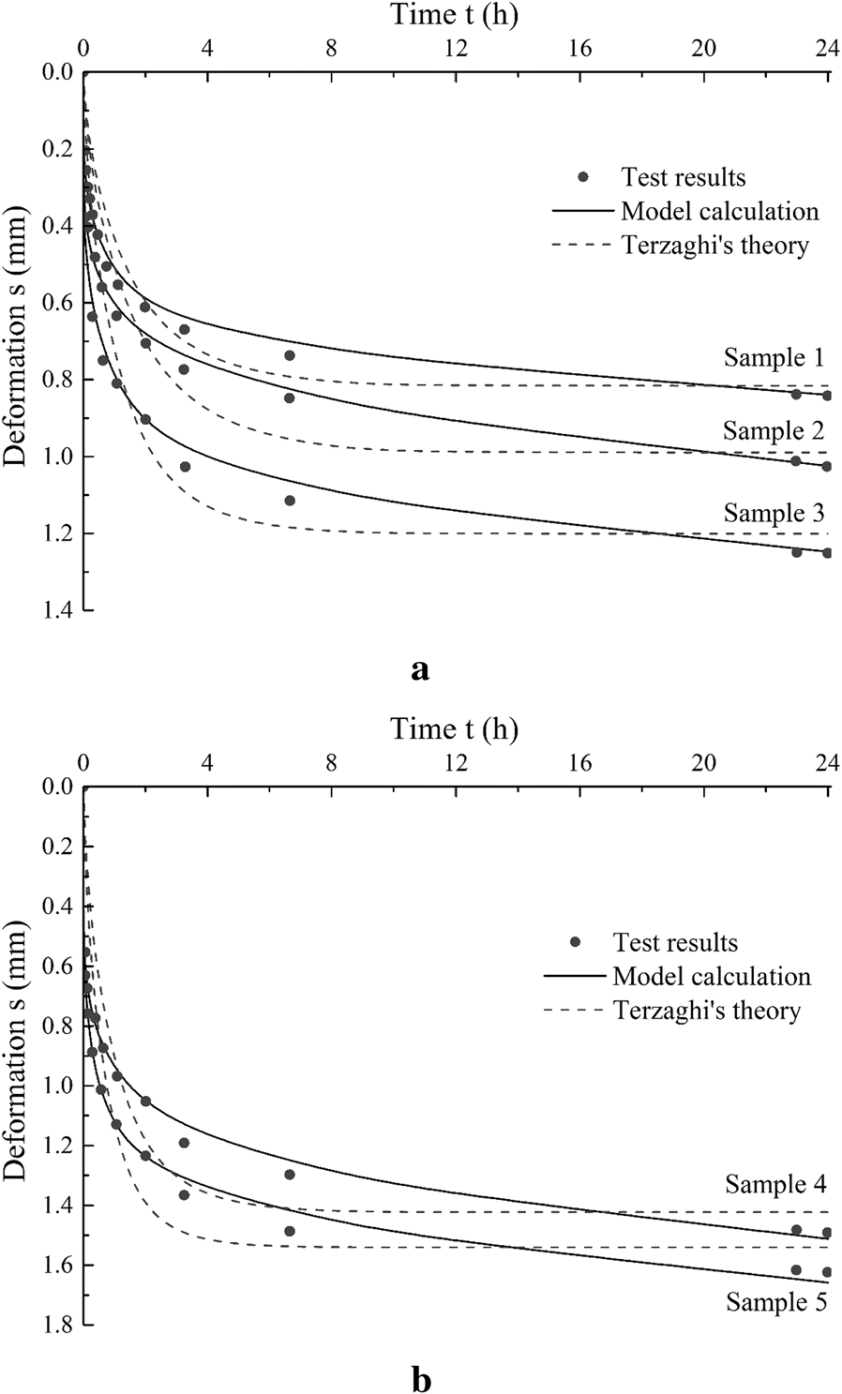
Modelling results compared with test results for peat samples in case 1, **a** samples 1, 2 and 3, **b** samples 4 and 5

The proposed consolidation model is used to simulate the test results, and calculation results are also compared with results from Terzaghi’s equation where the gas content is considered to be zero and solid matters are incompressible. The parameters used in the proposed model are listed in Table 2. The results are compared in Fig. 2. The figures show that, without considering the compressibility of the organic matters and gas bubbles, the consolidation curves predicted using Terzaghi’s equation are very smooth at the first few minutes, and the sudden settlement observed in the test results can not be fully captured, whilst the proposed model describes the test results quite well. Also the proposed model can describe the relatively large late stage deformation.

**Table 2:** Model parameters for peat samples in case 1

Sample no.	*σ* (kPa)	*e*	*e*_*g*_	*e*_*m*_	*E*_*m*_ (MPa)	*λ*	*S*_*g*_ (%)	*r*_0_ (μm)	*k* (m/s)
1	50	2.721	0.16	1.12	5.0	0.25	2.0	10	1.2 × 10^−10^
2	50	3.069	0.29	1.40	3.0	0.24	3.0	10	1.5 × 10^−10^
3	50	5.722	0.74	2.13	2.0	0.23	3.5	10	1.7 × 10^−10^
4	50	7.115	1.83	4.01	1.0	0.21	4.5	10	2.0 × 10^−10^
5	50	8.968	5.34	8.97	1.0	0.21	5.0	10	4.0 × 10^−10^

Table 2 shows that the gas content (*S*_*g*_) and the parameters of organic matter compression (*E*_*m*_, λ) obtained from the model are different in the five samples. In general, the gas content *S*_*g*_ increases and the compressive modulus *E*_*m*_ decreases with the increasing of organic matter content as shown in Tables 1 and 2. The time factor λ has a slight diminution with the increasing of organic matter content, but it keeps in a range of 0.21–0.25. The compression modulus of the organic matter *E*_*m*_ ranges from 1.0 to 5.0 MPa. The values are slightly higher than the initial constrained modulus *E*_0_ of Szczecin peat found by Meyer (1997). This may be due to the fact that the initial constrained modulus considers the compressibility of both peat and gas in peat samples. Gas content is within the range of 2–5 %, and the value increases with organic content and void ratio as shown in Table 2, but the conclusion on this can not be made based on this result.

### Case 2: Middleton peat from Wisconsin, USA

**Fig. 3:**
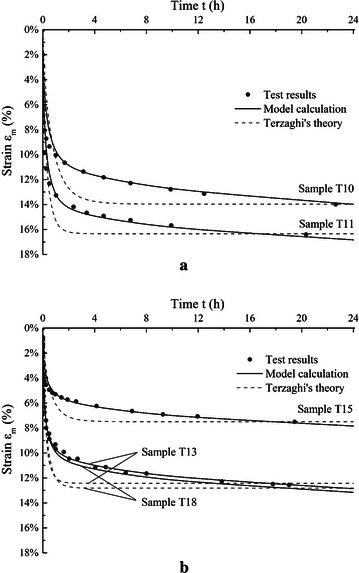
Modelling results compared with test results for peat samples in case 2, **a** samples T10 and T11, and **b** samples T13, T15, T18

Some typical studies and consolidation tests had been done by Mesri et al. (1997) on peat samples taken from Middleton, Wisconsin, USA. In their study, the authors mainly focused on some basic properties of peat including compression index *C*_*c*_ and secondary compression index *C*_*α*_. In all consolidation test results, immediate settlement had been observed but the authors mainly studied the secondary consolidation behavior of the material and the initial immediate settlement was not explained in the original publication. Five of the test results from Mesri et al. (1997) are analyzed using the proposed model. The basic properties are shown in Table 3. The model parameters are shown in Table 4. The model simulated deformation curves of each sample are compared with test results and results by Terzaghi’s equation in Fig. 3.

**Table 3:** Properties of peat samples in case 2

Parameters	*ρ* (g/cm^3^)	*γ*_*p*_ (kN/m^3^)	*ω*_*m*_ (%)	*ω* (%)	*e*
Values	0.96	15.9	92.7	740	12.224

**Table 4:** Model parameters for peat samples in case 2

Sample no.	*σ* (kPa)	*e*	*e*_*g*_	*e*_*m*_	*E*_*m*_ (MPa)	*λ*	*S*_*g*_ (%)	*r*_0_ (μm)	*k* (m/s)
T10	41	12.224	11.53	20.80	0.8	0.24	4.0	10	6.0 × 10^−10^
T11	96	12.224	11.53	20.80	1.1	0.20	4.0	10	6.0 × 10^−10^
T13	96	12.224	11.53	20.80	1.0	0.20	4.0	10	6.0 × 10^−10^
T15	30	12.224	11.53	20.80	0.8	0.24	4.0	10	6.0 × 10^−10^
T18	90	12.224	11.53	20.80	1.1	0.20	4.0	10	6.0 × 10^−10^

The compression modulus *E*_*m*_ of the organic matter obtained for the peat samples is relatively consistent comparing with that of the peat samples in case 1. This may due to the fact that the peat samples used in Mesri et al. (1997) are from a 2.5 m by 2.5 m test pit, which suggests that the variation of the properties in the peat samples could be less comparing to the samples used by Lv et al. (2011) where the peat samples are from a relatively larger area. The peat samples used in Mesri et al. (1997) have the same organic matter content around 92.7 %, on the contrary the 5 peat samples used by Lv et al. (2011) have an organic matter content range of 36.34–85.36 %. The time factor λ in this case has similar values with peat samples in case 1. What need to be noted in Table 4 is that the values of *E*_*m*_ and λ of samples T10 and T13 are a little larger than others. This may be due to the different loading levels applied on the samples. The vertical stress on samples T10 and T13 (30–41 kPa) are smaller than others (90–96 kPa). Apart from that, the parameters of the organic matter compression from the two cases are in a similar range.

In above 2 cases, the theoretical predictions of deformation or strain for peat samples show very close agreement with test results. The figures show that the proposed model is suitable for peat. For the deformation or strain curves of peat, an obvious initial deformation appears in a relative short time during the initial loading period. Then the strain rate tends to be slow with gradually completion of primary consolidation. But a significant deformation still develops during the following consolidation process. Plausible explanations of these phenomena are offered and mathematical treatments have been given in our model.

As mentioned, the author has done some preliminary studies on Victorian brown coal from Latrobe Valley, Australia. The compression modulus *E*_*m*_ of peat organic matters obtained from the calculation is much lower than the values of coal (about 30 MPa). That is because peat samples are normally consolidated but brown coal in the Latrobe Valley is highly over consolidated with overconsolidation ratio of 10 or above at the depth where the samples been taken. Therefore, the fibrous structure in peat is much more compressive comparing to coal grains. The time factor *λ* for peat obtained from the model is also higher than that of brown coal (0.042). This may due to the fact that the hollow structure in the fibers of peat samples are still well maintained as shown in Mesri et al. (1997), and the deformation of the hollow fibers may contribute to the creep of the organic particles which results in higher *λ* values. Although similar properties have been found in both peat and brown coal, different model values are obtained for the two materials. This may mainly due to the geological history, material structure and different basic indexes. Brown coal is usually fossilized from peat after a long time of coalification process. Peat has a lower density and higher void ratio and water content than brown coal.

## Discussion on model parameters

### Volume ratio of gas *e*_*g*_

The volume ratio of gas *e*_*g*_ represents the volume percentage of gas in peat samples. To study the parameter, *e*_*g*_ is normalized by 1 + *a*, which gives the gas volume content *S*_*g*_:

25 $$S_{g} = \frac{{e_{g} }}{1 + a}$$

A value range of 2–5 % for *S*_*g*_ is obtained in the model calculation. Literature reviewing shows that peat from field usually has a gas volume content of around 5–11 % (Hobbs 1986; Mesri et al. 1997). Considering peat samples are usually water saturated for a short time before testing, so a value range of 2–5 % for *S*_*g*_ is reasonable in the calculation.

### Volume ratio of organic matter e_m_

As the same definition of *e*_*g*_, the volume ratio of organic matter *e*_*m*_ represents the volume percentage of organic matter in peat samples. Normalizing *e*_*m*_ by 1 + *a*, we can get the organic matter volume content *S*_*m*_:

26 $$S_{m} = \frac{{e_{m} }}{1 + a}$$

**Fig. 4:**
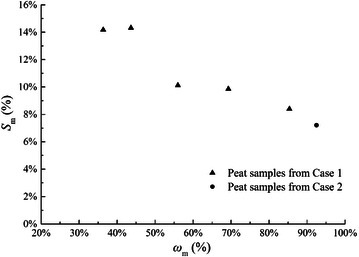
Relation between *S*_*m*_ and *ω*_*m*_

In fact, *e*_*m*_ or *S*_*m*_ is a kind of intrinsic parameter of peat which is directly decided by traditional organic matter content *ω*_*m*_, which is defined as the ratio of the mass of organic matter to the mass of total solid matter. The relationship between *S*_*m*_ and *ω*_*m*_ are shown in Fig. 4 for all the peat samples in above 2 case studies. It can be seen that the organic matter volume content *S*_*m*_ decreases with increasing of organic matter content *ω*_*m*_. The variation tendency is reasonable because when *ω*_*m*_ is high peat usually has high values of void ratio and water content, and the main space of a peat sample will be filled by water. Then the absolute value of volume for organic matter will be smaller. On the contrary, when organic matter content *ω*_*m*_ is low peat usually has a low water content, which means the solid phase can take a high volume percentage of a peat sample. So the organic matter volume content *S*_*m*_ can be larger when the organic matter content *ω*_*m*_ is low.

### Time factor λ

Parameter *λ* is a time factor in proposed compression model of organic matter. A relatively stable value range of 0.20–0.25 for *λ* is obtained in model calculation. The value is acceptable with slight variation, which reflects the deformation properties of organic matter with time *t*. The slight variation of *λ* could be caused by reasons like different organic matter content of peat, geological history and applied stress levels.

### Initial constrained modulus E_m_

**Fig. 5:**
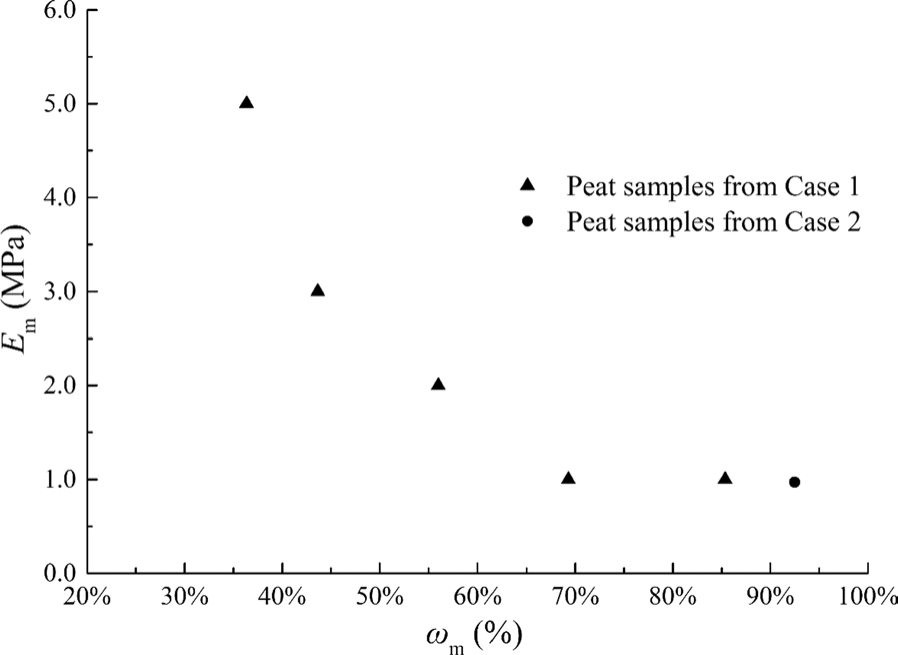
Relation between *E*_*m*_ and *ω*_*m*_

The initial constrained modulus *E*_*m*_ is another model parameter in proposed compression model of organic matter. The value of *E*_*m*_ varies in above 2 case studies, but it shows some regularity with organic matter content *ω*_*m*_. The obtained value relation between *E*_*m*_ and *ω*_*m*_ is shown in Fig. 5 for all the peat samples in the 2 case studies. It can be seen that *E*_*m*_ almost has a decreasing tendency with increasing of organic matter content *ω*_*m*_. This can be caused by the state of hollow structures and dense state of organic matter. Under conditions of low organic matter content, organic matter mixed with more minerals and can have a more dense state, which leads to a larger value of *E*_*m*_. Although a decreasing tendency between *E*_*m*_ and *ω*_*m*_ is obtained from Fig. 5, the conclusion can’t be simply made. Similar to parameter *λ*, the value of *E*_*m*_ could be effected by other reasons like geological history and applied stress levels.

## Effect of model parameters on consolidation curves

In above sections, a numerical model is established to study the consolidation behavior of peat containing organic matter and gas bubbles. In the model, the compression modulus *E*_*m*_ and time factor *λ* which describe the compression properties of organic matters, gas content *S*_*g*_ and initial gas bubble radius *r*_0_ are used. To study the effect of each parameter on the consolidation behavior of peat, sensitivity analyses are carried out in this section. In the analysis, only one of the 4 parameters is considered changing to calculate the consolidation curves. Taking peat sample 3 in case study 1 as an example in the following studies. What need to be noted is that the calculated curves are not all real for sample 3. The work mainly wants to show how the tendency of consolidation curves effected by a certain parameter.

### Effect of organic compression modulus

**Fig. 6:**
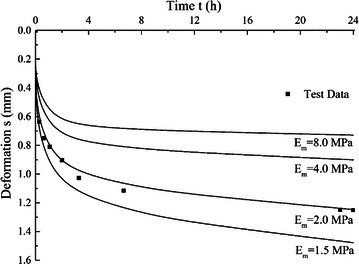
Effects of *E*_*m*_ on consolidation curves

For peat sample 3, different values of *E*_*m*_ with 1.2, 2, 4 and 8 MPa are used and other parameter values keep original and constant, *λ* is 0.23, *S*_*g*_ is 3.5 % and *r*_0_ is 10 μm. The calculated consolidation curves with different *E*_*m*_ values are shown in Fig. 6. The results show that total deformations of peat increase with decreasing *E*_*m*_. The initial deformation and the late stage of deformations are also larger with smaller *E*_*m*_ value.

### Effect of time factor

**Fig. 7:**
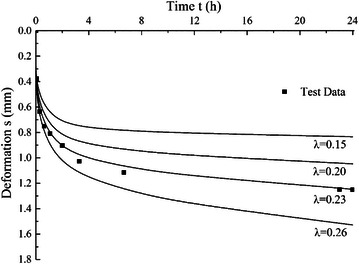
Effects of *λ* on consolidation curves

Same as above, different values of *λ* with 0.15, 0.20, 0.23 and 0.26 are used and other parameter values keep constant, *E*_*m*_ is 2 MPa, *S*_*g*_ is 3.5 % and *r*_0_ is 10 μm. The calculated consolidation curves with different *λ* values are shown in Fig. 7. The results show that larger *λ* values cause greater total deformation and parameter *λ* mainly has significant influence on late consolidation stage. Steeper consolidation curves can be obtained using larger *λ* values.

### Effect of gas content

**Fig. 8:**
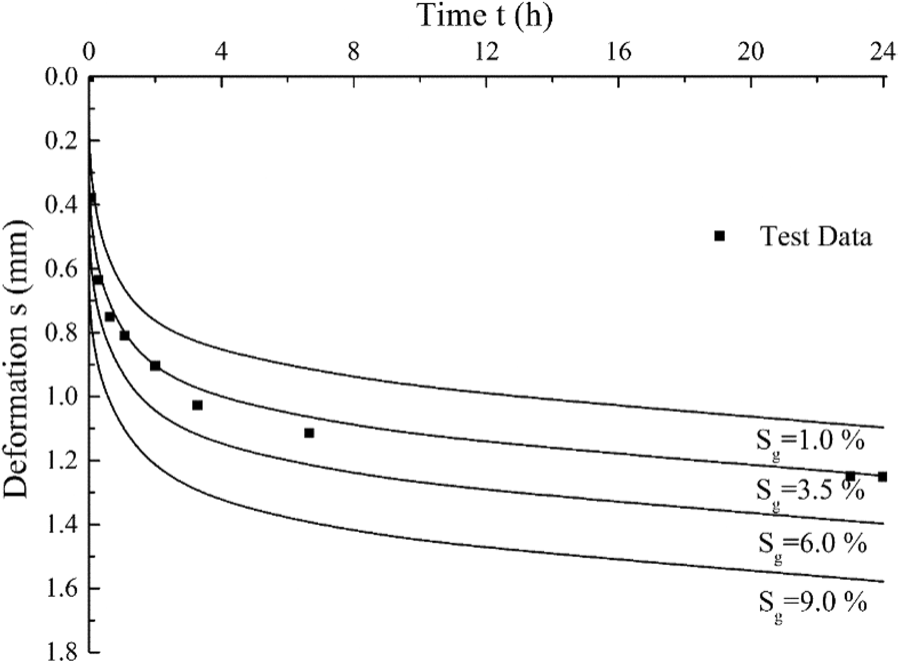
Effects of *S*_*g*_ on consolidation curves

To study the influence of gas content, different values of *S*_*g*_ with 1.0, 3.5, 6 and 9 % are used and other parameter values keep constant, *E*_*m*_ is 2 MPa, *λ* is 0.23 and *r*_0_ is 10 μm. The calculated consolidation curves with different *S*_*g*_ values are shown in Fig. 8. The results show that increasing of gas content *S*_*g*_ can cause the increase of total deformation, especially in the initial stage of consolidation. With gradually dissipation of excess pore water pressure, the strain increment rate of entrapped gas becomes smaller and doesn’t have significant influence on the late stage deformation, which leads to relatively parallel lines at late deformation stages as shown in Fig. 8.

### Effect of initial gas bubble radius

**Fig. 9:**
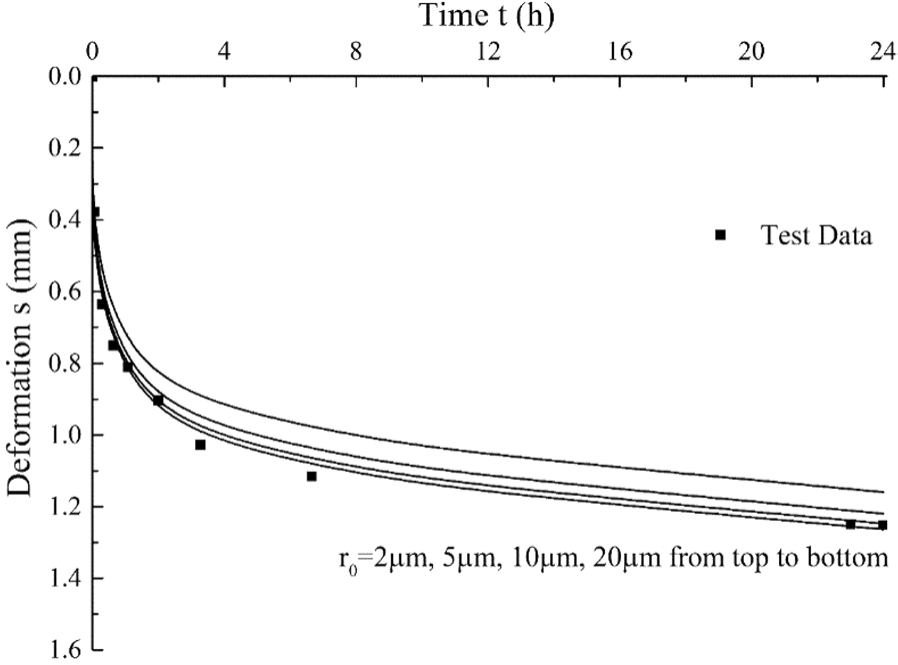
Effects of *r*_0_ on consolidation curves

**Fig. 10:**
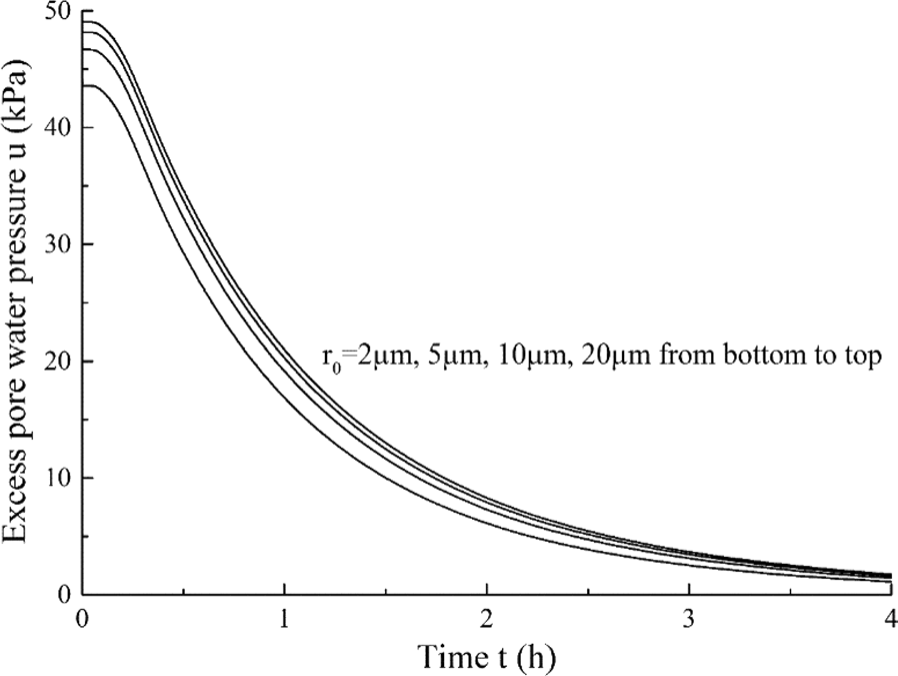
Effects of *r*_0_ on dissipation of excess pore water pressure

For gas bubble size, it mainly has influence on surface tension effect. Different values of *r*_0_ with 2, 5, 10 and 20 μm are used and other parameter values keep constant, *E*_*m*_ is 2 MPa, *λ* is 0.23 and *S*_*g*_ is 3.5 %. The calculated consolidation curves with different *r*_0_ values are shown in Fig. 9. The parameter *r*_0_ is different from the other three model parameters. It indicates the effect of surface tension on deformation and dissipation of excess pore water pressure. The results show that the influence of initial gas bubble radius *r*_0_ is not as significant as other parameters, especially under a larger value of *r*_0_ the surface tension effect can be almost neglected. In fact, the effect of *r*_0_ on consolidation curve is mainly by influencing the excess pore water pressure. The excess pore water pressure of the first 4 h on the midplane of peat sample under different *r*_0_ are shown in Fig. 10. The initial excess pore water pressure and the dissipation rate are both smaller under small gas bubble size, which decreases the amount of water drainage when other parameters keep constant. Therefore, smaller values of *r*_0_ can cause relatively remarkable influence on deformation of peat samples. On the other side, when larger gas bubbles are considered the surface tension effect can be neglected and the pore water pressure and pore air pressure can be assumed to be the same.

## Conclusion

Peat has special natural characteristics and engineering properties due to the high content of organic matter. The composition and structure of peat are complicated, and organic matter in peat can be compressible. Biogas generates from the natural deposits of peat and some will be trapped as small gas bubbles. Peat has special three phase composition containing gas bubbles, water, compressible organic matters and incompressible minerals. A consolidation model is proposed to study the deformation behavior of peat. The deformation of peat is considered as a coupling process of the volume change of gas bubbles, compression of organic matter and the drainage of water. Then the application of the model is carried out on some historical test data of peat by fitting the experimental results with the modelled consolidation curves.

The results show that the proposed model can well capture the unique consolidation behavior of peat. The gas content and the compression parameters of organic matter can be obtained using the model. Based on the experimental and numerical modelling results, conclusions can be obtained:

1: The obvious initial settlement observed in peat samples is due to the existence of gas bubbles and the compressibility of organic matters. The proposed model can be used to simulate this process, and as a result the gas content and compression parameters of the organic matter can be obtained by fitting the experimental and modeled results.

2: Based on the modeled results of the samples, the compression modulus of organic matter in peat is in a range of 0.8–5.0 MPa. The large range variation of this value is probably due to the organic matter content. The creep effect observed in peat at the late stage of the consolidation tests can be modelled with the stress–strain–time model of organic matter by introducing a time factor. The late stage deformation in peat is relatively large due to the presence of organic matter, which has been reflected by a large value of time factor in around 0.20–0.25 for the peat samples.

3: The mechanism of gas is very complicated in practical situation. In the proposed consolidation model, gas bubbles are simply assumed to follow Boyle’s law with considering the surface tension effect. Increasing gas content can cause larger settlements of peat and the surface tension effect can not be neglected when considering small gas bubble sizes.

The proposed model could be used in analyzing the consolidation behavior of peat, which contains both gas bubbles and compressible organic matters. It has the potential to be used for modelling compression behavior of similar engineering materials, for example brown coal.
